# *In situ* constructed oxygen-vacancy-rich MoO_3−*x*_/porous g-C_3_N_4_ heterojunction for synergistically enhanced photocatalytic H_2_ evolution†

**DOI:** 10.1039/d1ra05620d

**Published:** 2021-09-22

**Authors:** Yufeng Pan, Bin Xiong, Zha Li, Yan Wu, Chunjie Yan, Huaibin Song

**Affiliations:** Engineering Research Center of Nano-Geomaterials of Ministry of Education, Faculty of Materials Science and Chemistry, China University of Geosciences Wuhan 430074 China songhb@cug.edu.cn; Wuhan National Laboratory for Optoelectronics, Huazhong University of Science and Technology Wuhan Hubei 430074 P. R. China

## Abstract

A simple method was developed for enhanced synergistic photocatalytic hydrogen evolution by *in situ* constructing of oxygen-vacancy-rich MoO_3−*x*_/porous g-C_3_N_4_ heterojunctions. Introduction of a MoO_3−*x*_ precursor (Mo(OH)_6_) solution into g-C_3_N_4_ nanosheets helped to form a porous structure, and nano-sized oxygen-vacancy-rich MoO_3−*x*_*in situ* grew and formed a heterojunction with g-C_3_N_4_, favorable for charge separation and photocatalytic hydrogen evolution (HER). Optimizing the content of the MoO_3−*x*_ precursor in the composite leads to a maximum photocatalytic H_2_ evolution rate of 4694.3 μmol g^−1^ h^−1^, which is approximately 4 times higher of that of pure g-C_3_N_4_ (1220.1 μmol g^−1^ h^−1^). The presence of oxygen vacancies (OVs) could give rise to electron-rich metal sites. High porosity induced more active sites on the pores' edges. Both synergistically enhanced the photocatalytic HER performance. Our study not only presented a facile method to form nano-sized heterojunctions, but also to introduce more active sites by high porosity and efficient charge separation from OVs.

## Abstract 2

*In situ* growth method to construct a nano-sized oxygen-vacancy-rich MoO_3−*x*_/porous g-C_3_N_4_ heterojunction. MoO_3−*x*_ derived OV traps and porous g-C_3_N_4_ nanosheet derived short migration distance and plentiful edge active sites.

## Graphical abstract

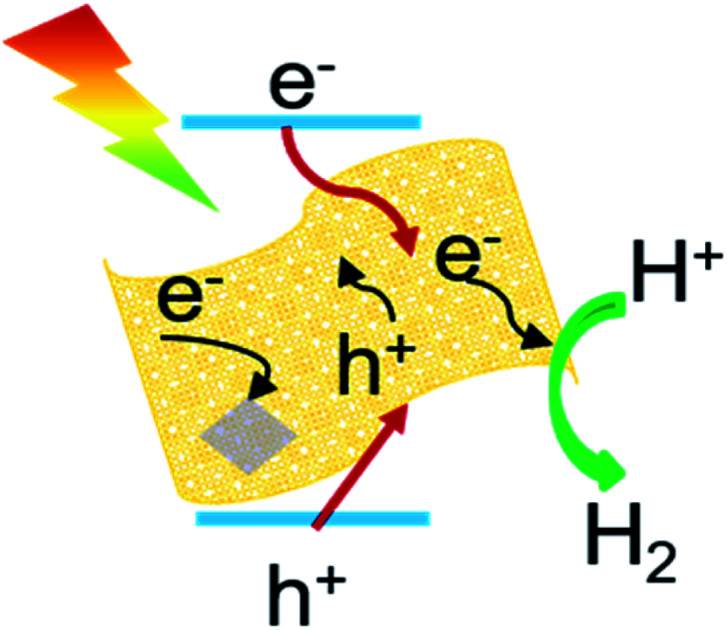

## 1. Introduction

Photocatalytic hydrogen evolution (HER) is considered to be one of the most promising ways to alleviate the environment and energy crisis.^1–6^ However, exploring highly efficient and environmentally-friendly catalysts remains a challenge for the current photocatalysis field.^7^ The efficiency of photocatalytic HER lies in three main aspects: (i) the generation and separation of photo-generated charges, (ii) the migration distance of carriers, and (iii) the oxidation–reduction reaction on the surface of photocatalysts.^8^ Various strategies have been proposed to improve the photocatalytic efficiency, with a broad range of photocatalysts.^7^

The prevailing photocatalysts include metal oxides,^9^ metal sulfides,^10^ metal nitrides,^11^ organometallic complexes,^12^ and metal-free semiconductors.^13–17^ Among the various photocatalysts, g-C_3_N_4_, a metal-free semiconductor, has attracted extensive attention in photocatalytic HER due to its outstanding characteristics, such as appropriate band edge, environmental friendliness, high thermal and chemical stability, facile fabrication and cost-effectiveness.^18,19^ However, the photocatalytic HER efficiency of g-C_3_N_4_ is still far from satisfactory, due to the low specific surface area, limited active sites and fast recombination rate of photogenerated electron–hole (e^−^–h^+^) charges.^20^ g-C_3_N_4_ nanosheets were reported previously with better performance than bulk g-C_3_N_4_ with increased surface area, higher photogenerated electron reduction potential, better electron transport capacity and longer lifetime *etc.* Our strategy has designed a more efficient nano-junction based on g-C_3_N_4_ nanosheets.

Based on the review, various strategies have been proposed based on g-C_3_N_4_, including electronic structure modulation, crystal structure engineering, nanostructure and hetero-structure construction.^8,21–25^ The construction of g-C_3_N_4_-based heterojunction can help the separation of charges at the interface, as a variety of g-C_3_N_4_-based heterojunctions has been reported, such as TiO_2_/g-C_3_N_4_,^26,27^ ZnO/g-C_3_N_4_,^28,29^ WO_3_/g-C_3_N_4_,^30^ WS_2_/g-C_3_N_4_,^31^ NiO/g-C_3_N_4_,^32^ ZnIn_2_S_4_/g-C_3_N_4_,^33^ Zn_*x*_Cd_1−*x*_In_2_S_4_/g-C_3_N_4_,^34^ Bi_2_Se_3_/g-C_3_N_4_,^35^ MoO_3_/1T-MoS_2_/g-C_3_N_4_,^36^*etc.* MoO_3_ has a large band gap of 3 eV and a high dielectric constant of 6–18, suitable for constructing heterojunction photocatalysis with g-C_3_N_4_.^37–39^ Oxygen-vacancy-rich MoO_3−*x*_ was even more favourable. The synthesis of porous g-C_3_N_4_ and few-layered MoO_3_ has been widely reported, however, the combination of porous g-C_3_N_4_ and oxygen-vacancy-rich MoO_3−*x*_ to construct MoO_3−*x*_/g-C_3_N_4_ photocatalytic system remains a great challenge. The introduction of porous structure in g-C_3_N_4_ usually produces many photocatalytic active sites.^40^ Xiao *et al.* introduced a bottom-up method for preparing porous few-layer g-C_3_N_4_ by a sequential molecule self-assembly, alcohol molecules intercalation, thermal-induced exfoliation and polycondensation process.^40^ Moreover, conventional silicon dioxide (SiO_2_) template or ammonium bicarbonate (NH_4_HCO_3_) has also been used as pore forming agent to prepare porous g-C_3_N_4_ in the process of polycondensation.^41,42^ For the construction of oxygen vacancies, metal oxides with oxygen vacancies are usually obtained by using hydrogen thermal treatment,^43^ high energy particle bombardment,^44^ and heating metal oxides under vacuum or oxygen depleted conditions.^45^ Although these methods have their own advantages, they are inevitably limited by high-energy input and complex post-processing. According to the literature, there are electrostatic composite and *in situ* construction method.^41,46^ The former is simple to implement, but it is greatly affected by the pH of photocatalytic reaction. Therefore, it is urgent to develop a new method to integrate porous structure and oxygen vacancy into a heterojunction system.

Herein, we propose a salt assisted *in situ* growth method to construct nano-sized oxygen-vacancy-rich MoO_3−*x*_/porous g-C_3_N_4_ heterojunction. The introduction of MoO_3−*x*_ precursor (Mo(OH)_6_) into g-C_3_N_4_ not only helps to prepare porous nanosheets, but also *in situ* grown oxygen-vacancy-rich MoO_3−*x*_ can form an atomic-scale compact heterointerface with g-C_3_N_4_. In additional, the heterojunction between MoO_3−*x*_ and g-C_3_N_4_ was established, which synergically improved photocatalytic HER performance, including higher charge separation efficiency, shorter charge transport path, and more catalytic active sites. By HR-TEM, HAADF-STEM and AFM, the morphology of the nanosized MoO_3−*x*_/porous g-C_3_N_4_ heterojunction was confirmed. XPS proved the oxygen-vacancy in MoO_3−*x*_. As a result, the optimized MoO_3−*x*_/g-C_3_N_4_ heterojunction produces H_2_ at 4694.3 μmol g^−1^ h^−1^, approximately 4 times higher of that of pure g-C_3_N_4_ (1220.1 μmol g^−1^ h^−1^). Our strategy can be universal for constructing diverse heterojunction with OVs and porous characteristics.

## 2. Experimental sections

### 2.1. Materials and reagents

All chemical reagents and materials were purchased and used without further purification. Molybdenum powder (Mo, 98%), urea ((NH_2_)_2_CO, ≥98%) were purchased from Sigma. Sodium chloride (NaCl, AR), absolute ethanol (CH_3_CH_2_OH, AR), and triethanolamine (TEOA, AR) were purchased from Sinopharm Chemical Reagent Co., Ltd. All chemicals were analytical grade and used without further purification.

### 2.2. Preparation of g-C_3_N_4_

g-C_3_N_4_ was synthesized by thermal condensation of urea directly. In detail, 10 g urea powder was placed in an alumina crucible and heated to 550 °C for 2 h in static air with a heating rate of 5 °C min^−1^. The resulting yellow agglomerates were then collected and milled into powder for further synthesis and measurements.

### 2.3. Preparation of Mo(OH)_6_ precursor

Typically, 0.1 g molybdenum (Mo) powder was dispersed in 10 mL ethanol with stirring for several minutes. Then 0.35 mL H_2_O_2_ (30%) solution was added into the Mo power suspension solution. After 18 h, the Mo oxide solution turned from grey to yellow and finally turned to blue.

### 2.4. Preparation of MoO_3_

The 10 mL Mo(OH)_6_ precursor was dried in a 60 °C vacuum oven for 30 min, then transferred to a tubular furnace and calcined at 400 °C for 2 h (5 °C min^−1^, under nitrogen condition).

### 2.5. Preparation of MoO_3−*x*_

The Mo(OH)_6_ precursor was added into a certain amount of NaCl, stirred well, and dried on the heating mantle at 80 °C. Then, transferred to a quartz tube furnace, to anneal for 2 h at 280 °C (5 °C min^−1^, under nitrogen condition). The sample was dissolved in deionized water, and then filtered, washed and dried to obtain solid powder.

### 2.6. Preparation of MoO_3−*x*_/porous g-C_3_N_4_ nanosheets

A typical fabrication process as follows: 50 mg g-C_3_N_4_ was dissolved in 200 mL of anhydrous ethanol and dispersed it evenly by ultrasonic treatment. 200 g NaCl prepared beforehand was added to the solution, followed by stirring, and the ethanol was removed by placing it on a heating mantle at 80 °C, the obtained sample was annealed at 300 °C for 2 h in a quartz tube furnace (5 °C min^−1^, under nitrogen condition). The Mo(OH)_6_ precursor was added into the above g-C_3_N_4_/NaCl mixture, stirred well, and dried on the heating table at 80 °C. The dried powder was transferred to a quartz tube furnace, to anneal for 2 h at 280 °C (5 °C min^−1^, under nitrogen condition). The obtained sample was dissolved in deionized water, filtered, washed and dried to obtain solid powder. 1%, 5%, 10%, 50% MoO_3−*x*_/g-C_3_N_4_ samples were prepared by adjusting the mass ratio of Mo powder to g-C_3_N_4_. NaCl acts as a removable template, provides a place for the construction of heterojunction *in situ*, and plays a good role in dispersing g-C_3_N_4_ precursor, so that Mo(OH)_6_ can better contact with g-C_3_N_4_. As an antisolvent, ethanol does not dissolve NaCl and can be quickly removed from the system under 80 °C heating, so it acts as a medium for mixing the two materials.

### 2.7. Characterization

The phase structures of samples were recorded by X-ray powder diffraction (XRD, BrokerAXS Germany). Chemical state analysis was performed by using X-ray photoelectron spectroscopy (XPS, ESCALab 250). FTIR spectrometer (Nicolet iS50) was applied to investigate the functional groups and chemical structure of samples. Scanning electron microscope (SEM, HITACHI SU8010 Hitachi Japan), transmission electron microscope (TEM, Tecnai G20) and atomic force microscope (AFM, BRUKER MultiMode 8) were conducted to confirm the morphology and thickness of samples. Ultraviolet-visible (UV-vis) spectrophotometer (Shimadzu instruments Ltd., Suzhou, UV-2600) and Spectrofluorometer (Edinburgh Instruments Ltd., FS5) measurements were performed to study optical properties of samples. Photocurrent response, EIS Nyquist plots and Mott–Schottky measurements were conducted to comprehend electrochemical properties with the help of CHI 760E electrochemical system (Chenhua, Shanghai, China).

### 2.8. Photoelectrochemical measurement

Photoelectrochemical experiments were carried out on the CHI 760E electrochemical workstation using a traditional three-electrode system. The Ag/AgCl electrode was used as the reference electrode, Pt electrode was used as the counter electrode, and photocatalyst/ITO was used as working electrode. The detailed preparation of working electrode is as follows: firstly, 10 mg catalyst was dispersed in 2 mL DI water, followed by another ultra-sonication for half an hour to form a uniform suspension. Secondly, the suspension was dropped onto the surface of the ITO glass. Finally, the ITO glass was placed under an infrared lamp for drying. 0.1 mol L^−1^ sodium sulfate solution and a 300 W xenon lamp were used as electrolyte solution and light source respectively, in the photocurrent responses and electrochemical impedance spectra experiments. In Mott–Schottky measurement, the frequency is 1000 Hz, the potential range is −1 to 1 V, and the electrolyte solution is 0.5 mol L^−1^ sodium sulfate solution.

### 2.9. Photocatalytic H_2_ evolution test

The photocatalytic H_2_ evolution experiments were carried out in a 200 mL Pyrex reaction cell connected to a glass closed gas circulation system with vacuum (Labsolar-III (AG), Perfectlight Technology Co. Ltd, Beijing, P. R. China). In a photocatalytic reaction, 50 mg as-prepared photocatalyst powder was suspended in a glass cylindrical reactor, then, added to 90 mL deionized water, and stirred for a while. 10 mL triethanolamine (TEOA) was added in the above suspension as a sacrificial agent. The photocatalytic reaction was performed using a 300 W xenon lamp (*λ* = 320–780 nm). Before irradiation, the whole system maintains a vacuum to avoid contact with air. H_2_PtCl_6_ aqueous solution was added as the precursor for the co-catalyst Pt, which was *in situ* photo-reduced during the photocatalytic reaction (2 wt% Pt). The amount of H_2_ produced during the photocatalytic reaction was determined by a gas chromatography (Techcomp, GC7900) (TCD detector, 5 Å molecular sieve column) for every one hour.

## 3. Results and discussion

### 3.1. Synthesis of nano-sized oxygen-vacancy-rich MoO_3−*x*_/porous g-C_3_N_4_ heterojunction by salt assisted *in situ* growth method

**Fig. 1:**
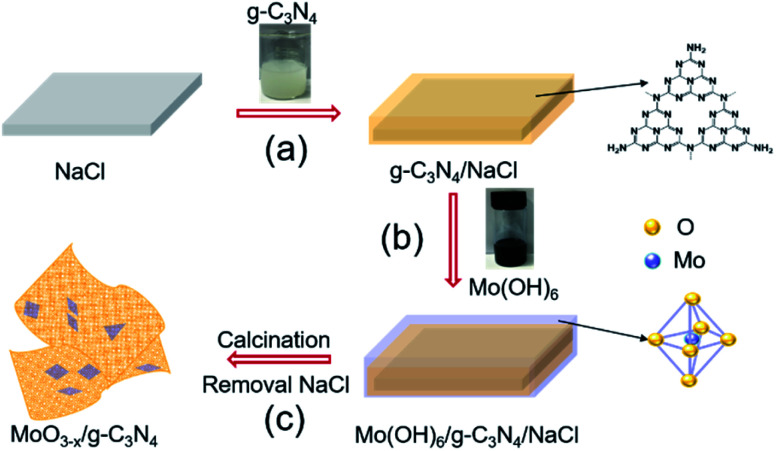
Schematic diagram of the fabrication processes of oxygen-vacancy-rich MoO_3−*x*_/porous g-C_3_N_4_ heterojunction.

We schematically illustrated the method in Fig. 1: firstly, g-C_3_N_4_ nanosheets were synthesized as the method reported and dispersed in anhydrous ethanol by ultrasonication, followed by adding NaCl powder. After the removal of ethanol by annealing at 80 °C, g-C_3_N_4_ nanosheets/NaCl was obtained (step 1). Secondly, the ethanol solution of Mo(OH)_6_ as precursor was mixed with the prepared g-C_3_N_4_ nanosheets/NaCl, annealing at 280 °C in a CVD furnace to obtain MoO_3−*x*_/g-C_3_N_4_/NaCl (step 2). Washing with deionized water several times to remove NaCl, the MoO_3−*x*_/g-C_3_N_4_ heterojunction material was obtained (step 3). In addition, by changing the mass ratio of Mo powder to g-C_3_N_4_ (1%, 5%, 10%, and 50%), MoO_3−*x*_/g-C_3_N_4_ samples with different Mo percentage were also prepared. The amorphous structure of prepared MoO_3−*x*_/g-C_3_N_4_ nanosheets was confirmed by X-ray diffraction (XRD) analysis as shown in Fig. S1.† Fig. S2† shows the XRD pattern of pure α-MoO_3_ fabricated from Mo(OH)_6_ precursor.

### 3.2. Morphological analysis

**Fig. 2:**
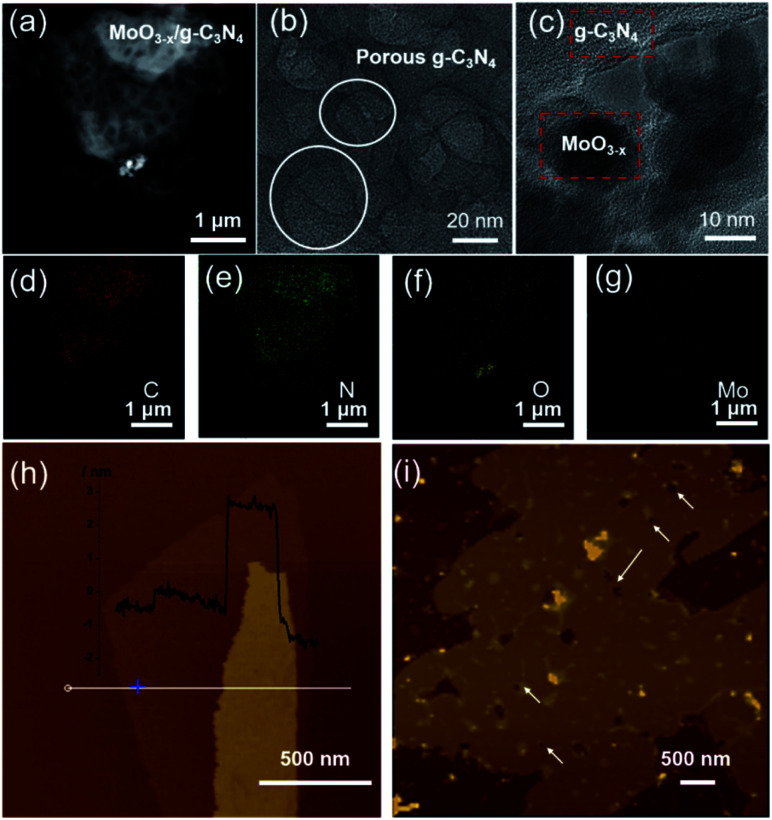
(a) HAADF-STEM image. (b and c) TEM images. (d–g) EDX elemental maps of MoO_3−*x*_/porous g-C_3_N_4_ heterojunction (C, N, O, Mo). (h) AFM images of BCN and (i) MoO_3−*x*_/porous g-C_3_N_4_ heterojunction.

HAADF-STEM and TEM images revealed the abundant nano-sized pores in nanosheets with tens of nanometers diameter (Fig. 2a and b), consistent with SEM observation (Fig. S3†). MoO_3−*x*_ nanocrystals assembled on the surface of g-C_3_N_4_ nanosheets (Fig. 2c and S4†). For further confirmation, EDX elemental mapping of 5% MoO_3−*x*_/g-C_3_N_4_ nanosheets indicated C, N, O, Mo elements distribution in the heterojunction (Fig. 2d–g). Atomic force microscopy (AFM) images showed the thicknesses of 5% MoO_3−*x*_/g-C_3_N_4_ heterojunction around 3–5 nm and pore diameter around tens of nanometers, consistent with (Fig. 2h and S5†). Thus, the microscopic morphology of MoO_3−*x*_/porous g-C_3_N_4_ heterojunction has been confirmed.

### 3.3. The formation of porous g-C_3_N_4_ nanosheets

**Fig. 3:**
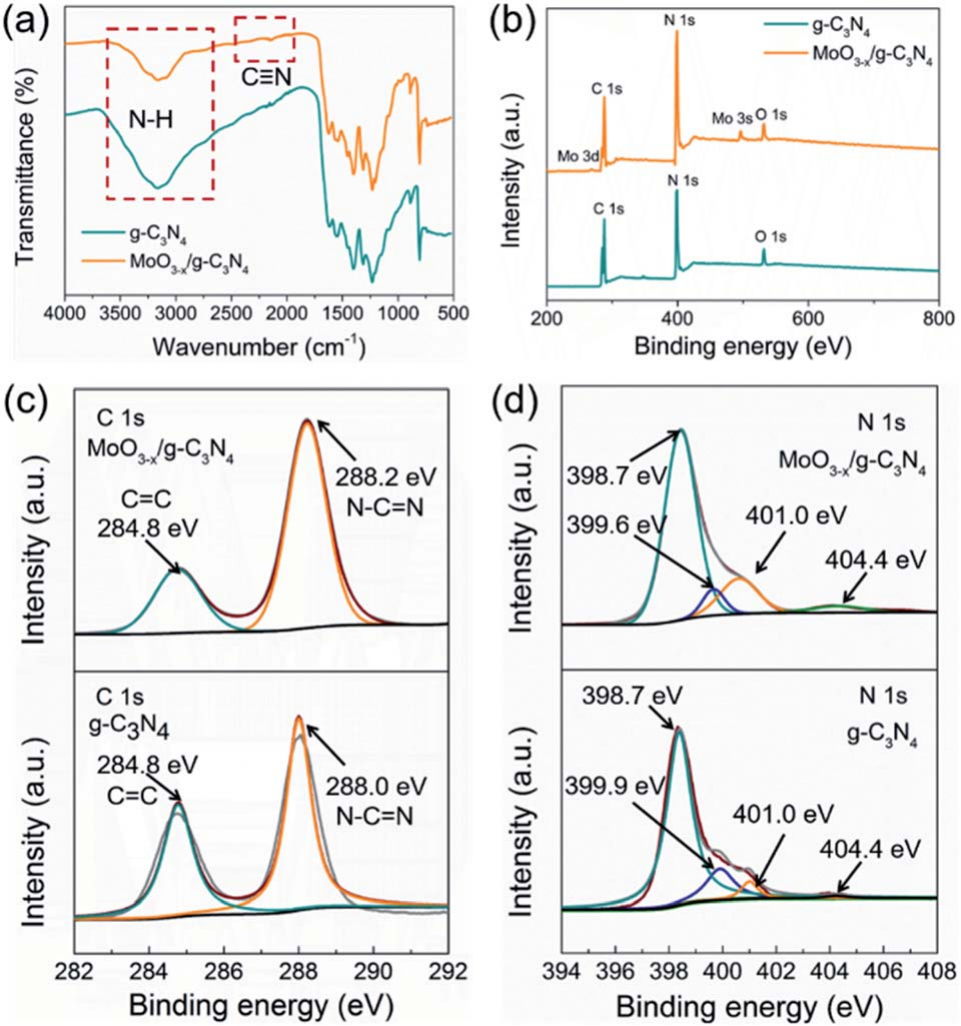
(a) FTIR spectrum, (b) XPS survey spectra, (c) C 1s XPS profiles and (d) N 1s XPS profiles of g-C_3_N_4_ and MoO_3−*x*_/g-C_3_N_4_ (5%) samples.

FT-IR was applied to analyse the chemical structures of g-C_3_N_4_ nanosheets and 5% MoO_3−*x*_/g-C_3_N_4_ (Fig. 3a). A new vibration band at 2178 cm^−1^ in MoO_3−*x*_/g-C_3_N_4_ nanosheets, attributed to C≡N bond, indicated the deprotonation of the amino group (–NH_2_) in g-C_3_N_4_ nanosheets.^8^ On the other side, NH_3_ was released in the deprotonation process, and led to the formation of pores.

To further investigate the chemical states and elemental composition of the samples, XPS and elemental analysis measurements were performed, respectively. As shown Fig. 3b, the XPS spectrum of 5% MoO_3−*x*_/g-C_3_N_4_ nanosheets exhibited C 1s and N 1s signals at the position same as that of g-C_3_N_4_ nanosheets. In Fig. 3c, the C 1s peak of g-C_3_N_4_ was fitted into two peaks at 284.8, and 288.0 eV, assigned to graphitic C–C, and sp^2^-hybridized carbon in N containing aromatic ring (N–C=N), respectively.^47^ The C 1s peak of MoO_3−*x*_/g-C_3_N_4_ (5%) nanosheets slightly shifted from 288.0 eV to 288.2 eV, attributed to the interaction between –NH_2_ and HO–Mo(OH)_5_.^8^ In Fig. 3d, the N 1s peak for g-C_3_N_4_ could be deconvoluted into four peaks at 398.7, 399.9, 401.0 and 404.4 eV, attributed to the sp^2^-bonded N in the tri-*s*-triazine units (C=N–C), bridging nitrogen atoms in N–(C)_3_, nitrogen atoms bonded with hydrogen atoms (–NH_*x*_) and the charging effects, respectively.^47^ Compared with g-C_3_N_4_ nanosheets, MoO_3−*x*_/g-C_3_N_4_ (5%) nanosheets showed obvious shift of N–(C)_3_ peaks (from 399.9 eV to 399.6 eV), suggesting cyano-groups and consistent with the FTIR results.^48^ In addition, the introduction of N defects can be supported by elemental analysis measurements as showed in Table S1,† supporting NH_3_ releasing. The N/C atomic ratio for g-C_3_N_4_ is 1.32, close to the ideal g-C_3_N_4_ composition (N/C = 1.33),^49^ while the N/C atomic ratios for MoO_3−*x*_/g-C_3_N_4_ (5%) decrease to 1.02, suggesting the loss of lattice nitrogen in MoO_3−*x*_/g-C_3_N_4_ (5%) due to the production of NH_3_.

### 3.4. The formation of oxygen vacancies on MoO_3−*x*_

**Fig. 4:**
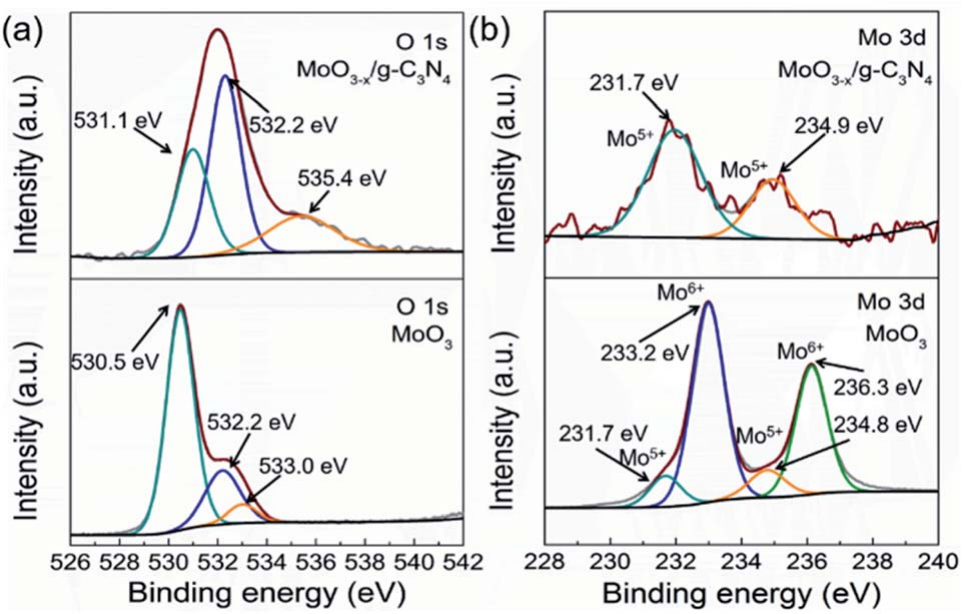
(a) O 1s XPS and Mo 3d XPS profiles (b) of α-MoO_3_ and MoO_3−*x*_/g-C_3_N_4_ (5%) nanosheets.

As a reference, we synthesized MoO_3_ by the reported method. We compared the XPS spectra of the MoO_3_ and 5% MoO_3−*x*_/g-C_3_N_4_ in Fig. 4a and b. For MoO_3_, the O 1s peaks at 530.5, 532.2, and 533.0 eV, are due to Mo–O, oxygen vacancy, and –OH, respectively.^50^ For MoO_3−*x*_/g-C_3_N_4_ (5%) nanosheets, the O 1s peaks from Mo–O and –OH positively shifted 0.6 and 1.6 eV, respectively, attributed to the interaction between the –OH of Mo(OH)_6_ and the –NH_2_ of g-C_3_N_4_. In order to further obtain more details information of oxygen vacancy, the ratios of different O species of MoO_3_ and MoO_3−*x*_/g-C_3_N_4_ (5%) calculated by XPS, the analysis results are shown in Tables S2 and S3.† The concentration of oxygen vacancy increased from 21.84% to 48.67% in MoO_3_ and MoO_3−*x*_/g-C_3_N_4_ (5%), respectively. By the O 1s spectra comparison, the ratio of Mo–O decreases and the ratio of –OH increases, suggesting the unsaturation of Mo coordination. In MoO_3−*x*_/g-C_3_N_4_ (5%) composite, the broadened doublets at 231.7 and 234.9 eV are ascribed to the electron binding energies of the Mo 3d_3/2_ and Mo 3d_5/2_ of Mo^5+^ (Fig. 3b). The existence of the Mo^5+^ may come from the oxygen vacancies and unsaturated coordination of Mo.^51,52^

### 3.5. Photocatalytic H_2_ evolution

**Fig. 5:**
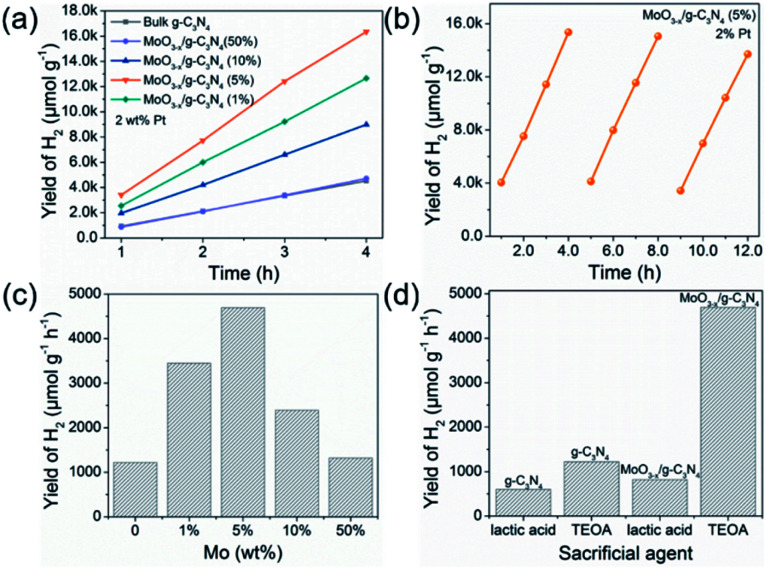
Photocatalytic H_2_ evolution performance and stability test. (a) The photocatalytic H_2_ evolution of MoO_3−*x*_/g-C_3_N_4_ samples with 2 wt% Pt as co-catalyst. (b) Photocatalytic cycle stability test of 5% MoO_3−*x*_/g-C_3_N_4_ with 2 wt% Pt as co-catalyst. (c) Optimum photocatalytic H_2_ evolution efficiency of MoO_3−*x*_/g-C_3_N_4_ photocatalysts with different contents of MoO_3−*x*_. (d) The photocatalytic hydrogen evolution performance for g-C_3_N_4_ and 5% MoO_3−*x*_/g-C_3_N_4_ with 2 wt% Pt as co-catalyst in different sacrificial agent systems (lactic acid and TEOA).

Fig. 5a exhibits HER rates of the as-prepared samples all with 2 wt% Pt as co-catalyst and TEOA as hole-sacrificial agent. The 5% MoO_3−*x*_/g-C_3_N_4_ heterojunction exhibits the highest HER activity as the optimized condition and lower or higher MoO_3−*x*_ percentage induced less HER activity. With the further increasing MoO_3−*x*_, excess MoO_3−*x*_ has a tendency to self-aggregate, reducing effective interfacial area between MoO_3−*x*_ and g-C_3_N_4_ and the active sites. So, the overmounted MoO_3−*x*_ led to lower performance. Fig. 5b shows that the high HER stability of 5% MoO_3−*x*_/g-C_3_N_4_ nanosheets, with no obvious decrease during 3 cycles. Fig. 5c compared the HER rate with different percentage of MoO_3−*x*_ in MoO_3−*x*_/g-C_3_N_4_ nanosheets. The 5% MoO_3−*x*_/g-C_3_N_4_ nanosheets exhibits the highest HER rate (4694.3 μmol g^−1^ h^−1^), about 4 times higher than pure g-C_3_N_4_ (1220.1 μmol g^−1^ h^−1^). Fig. 5d compared the HER rates of the pure g-C_3_N_4_ and 5% MoO_3−*x*_/g-C_3_N_4_ nanosheets using lactic acid and TEOA as hole-sacrificial agents, respectively. MoO_3−*x*_/g-C_3_N_4_ (5%) nanosheets exhibited higher HER rates in both systems of lactic acid and TEOA as hole-sacrificial agents. Photocatalytic hydrogen evolution rates of g-C_3_N_4_, 5% MoO_3_/g-C_3_N_4_ and 5% MoO_3−*x*_/g-C_3_N_4_ as shown in Fig. S6.† 5% MoO_3−*x*_/g-C_3_N_4_ showed the best HER performance, which further proved the promoting effect of OVs in MoO_3−*x*_/g-C_3_N_4_ photocatalyst on HER. We also compared the HER performance of similar photocatalysts reported in some recent references as shown in Table S4.†

### 3.6. The electronic structure and photocatalytic mechanism

**Fig. 6:**
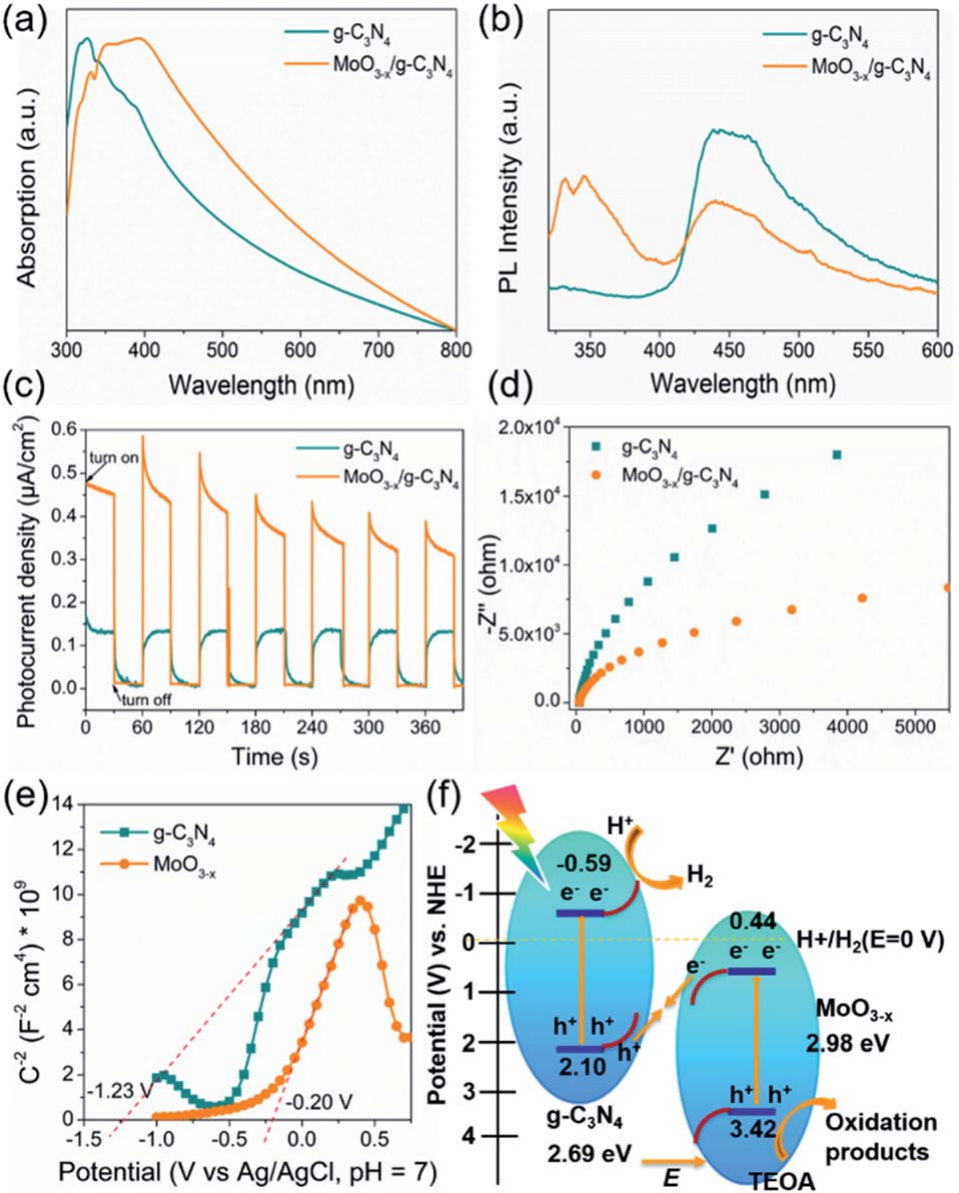
(a) UV-vis spectra of g-C_3_N_4_ and 5% MoO_3−*x*_/g-C_3_N_4_. (b) PL emission spectra of g-C_3_N_4_ and 5% MoO_3−*x*_/g-C_3_N_4_. (c) The transient photocurrent responses under dark and irradiated conditions. (d) The electrochemical impedance spectra of g-C_3_N_4_ and 5% MoO_3−*x*_/g-C_3_N_4_ photocathodes under light. (e) Mott–Schottky plots of g-C_3_N_4_ and MoO_3−*x*_. (f) The photocatalytic H_2_ evolution mechanism of the MoO_3−*x*_/g-C_3_N_4_ system under light irradiation (*λ* = 320–780 nm).

The optical absorption properties and band gap of the heterojunction were studied by UV-vis absorption spectroscopy. The g-C_3_N_4_ shows an absorption edge of approximately 435 nm (Fig. 6a), consistent with the ref. 53. The 5% MoO_3−*x*_/g-C_3_N_4_ heterojunction exhibits a red-shift of absorption edge, given to the interfacial charge transfer (Fig. 6a). UV-vis absorption spectra of g-C_3_N_4_ and MoO_3−*x*_ is shown in Fig. S7,† the illustrations are their corresponding Tauc's plot. In Fig. 6b, the pure g-C_3_N_4_ nanosheets in ethanol exhibited a strong PL peak at 450 nm, while 5% MoO_3−*x*_/g-C_3_N_4_ nanosheets with the same concentration of g-C_3_N_4_ nanosheets in ethanol showed much smaller PL peak at 450 nm. The PL peak at 350 nm was attributed to MoO_3−*x*_ (excitation wavelength: 300 nm). These results suggest that, in the heterojunction, the MoO_3−*x*_ absorbed the excitation energy and transferred some part to g-C_3_N_4_. In Fig. 6c, the 5% MoO_3−*x*_/g-C_3_N_4_ exhibit higher photocurrent density than g-C_3_N_4_. Compared with pure g-C_3_N_4_, 5% MoO_3−*x*_/g-C_3_N_4_ nanosheets have stronger photocurrent density as well as smaller arc radius (Fig. 6d), leading to lower resistance and favourable for charge separation. The corresponding optical band-gaps (*E*_g_) were calculated to be 2.69 eV for g-C_3_N_4_ and 2.98 eV for MoO_3−*x*_ (Fig. S7a and b†). In Mott–Schottky plots (Fig. 6e), the extrapolated conduction band edge (*E*_CB_) positions of g-C_3_N_4_ and MoO_3−*x*_ are −1.23 and −0.20 V (*vs.* Ag/AgCl, pH = 7) or −0.59 and +0.44 V (*vs.* NHE, pH = 0), respectively. Regarding *E*_g_ of g-C_3_N_4_ (2.69 eV) and MoO_3−*x*_ (2.98 eV) nanosheets, the valence band energy (*E*_VB_) was calculated as 2.10 and 3.42 eV (*vs.* NHE, pH = 0) as shown in Fig. 6f. According to the step-scheme photocatalytic system,^30^ the e^−^ on the CB of MoO_3−*x*_ would recombine with the h^+^ of VB g-C_3_N_4_ at the interface in the MoO_3−*x*_/g-C_3_N_4_. Consequently, e^−^ accumulated in the CB of g-C_3_N_4_ and h^+^ accumulated in the VB of MoO_3−*x*_. H^+^ was reduced to hydrogen by accumulated electrons at the CB of g-C_3_N_4_, while the TEOA was oxidized by the h^+^ accumulated at the VB of MoO_3−*x*_.

## 4. Conclusions

In this work, we have constructed oxygen-vacancy-rich MoO_3−*x*_/porous g-C_3_N_4_ nanosheets heterojunction by *in situ* growth. The heterojunction photocatalysts under light (*λ* = 320–780 nm) accelerated photogenerated e^−^–h^+^ separation and hydrogen evolution reaction. This method is simple and feasible, which endows g-C_3_N_4_ nanosheets with highly porous structure and more active sites, and endows MoO_3−*x*_ with oxygen-vacancy-rich MoO_3−*x*_ and more efficient charge separation, synergistically enhancing the photocatalytic performance. It could be a general method to inorganic semiconductor heterojunction or even multi-junction for photocatalysts.

## Author contributions

H. B. S. conceived the project. Y. F. P. designed and performed the experiments. H. B. S. and Y. F. P. analyzed and interpreted the experimental data. Y. F. P. wrote the paper. H. B. S., Y. W. and Z. L. revised the paper. All authors discussed the results and commented on the manuscript.

## Conflicts of interest

There are no conflicts to declare.

## Supplementary Material

RA-011-D1RA05620D-s001
