# Supplementary material for: In situ constructed oxygen-vacancy-rich MoO3−x/porous g-C3N4 heterojunction for synergistically enhanced photocatalytic H2 evolution
Source: RSC Adv. 2021 Sep 22;11(50):31219–25. doi: 10.1039/d1ra05620d (PMC9041325; doi:10.1039/d1ra05620d)
Supplement: RA-011-D1RA05620D-s001 [file RA-011-D1RA05620D-s001.pdf]

Electronic Supplementary Material (ESI) for RSC Advances.
This journal is © The Royal Society of Chemistry 2021

Electronic Supplementary Material (ESI) for RSC Advances.

Supplementary information

In situ Constructed Oxygen-vacancy-rich MoO_{3-x}/Porous *g*-C_3N_4 Heterojunction for Synergistically Enhanced Photocatalytic H_2 Evolution

Yufeng Pan,[a b] Bin Xiong,[a b] Zha Li,[c] Yan Wu,[a b] Chunjie Yan,[a b] Huaibin Song*[a b]

[a] Faculty of Materials Science and Chemistry, China University of Geosciences, Wuhan 430074, Hubei, P. R. China

[b] Engineering Research Center of Nano-Geomaterials of Ministry of Education, China University of Geosciences, Wuhan 430074, Hubei, P. R. China

[c] Wuhan National Laboratory for Optoelectronics, Huazhong University of Science and Technology, Wuhan, Hubei, 430074, P. R. China

* Corresponding author: Huaibin Song
E-mail: songhb@cug.edu.cn

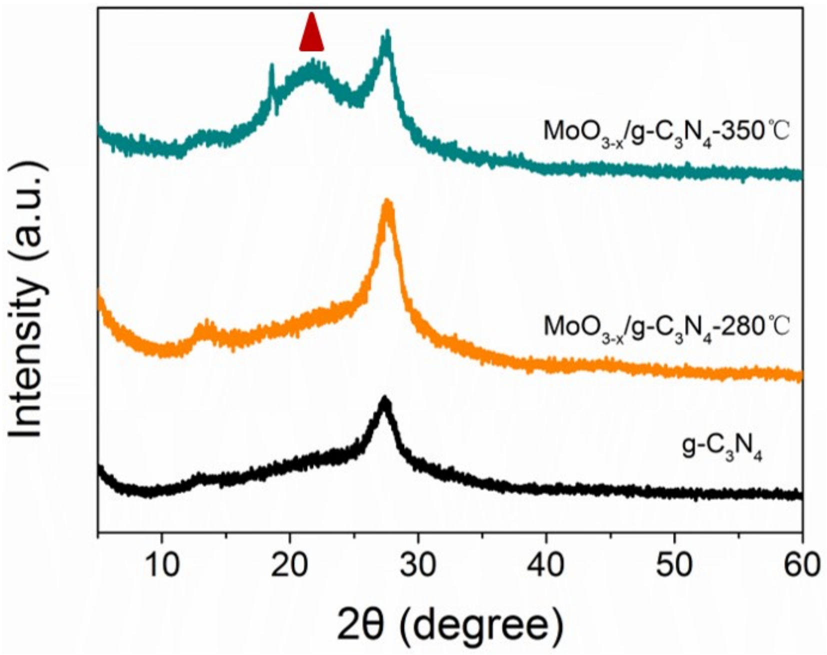

Figure S1 XRD patterns of *g*-C_3N_4, MoO_{3-x}/*g*-C_3N_4 (calcination at 280 °C), and MoO_{3-x}/*g*-C_3N_4 (calcination at 350 °C).

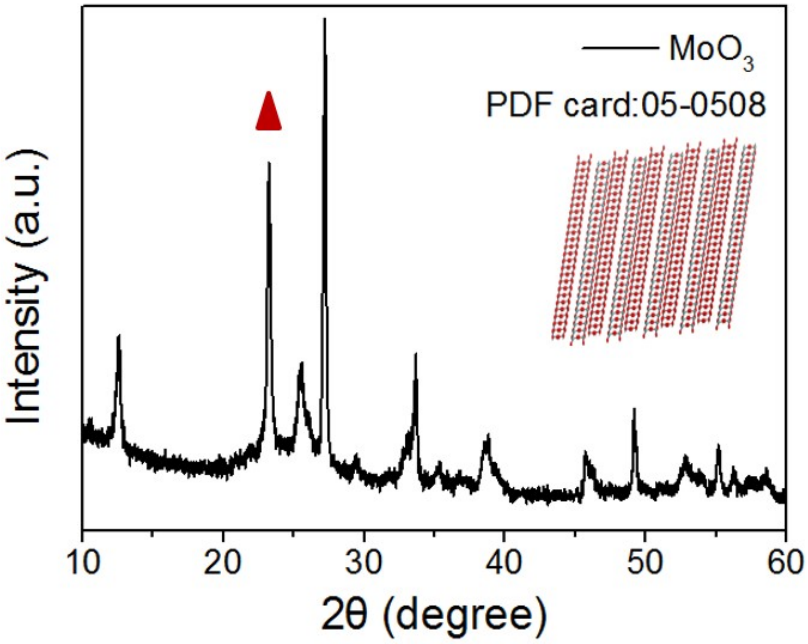

Figure S2 XRD pattern of MoO_3 (The Mo $(OH)_6$ precursor was calcined at 400 ℃), the illustration shows the crystal structure of MoO_3.

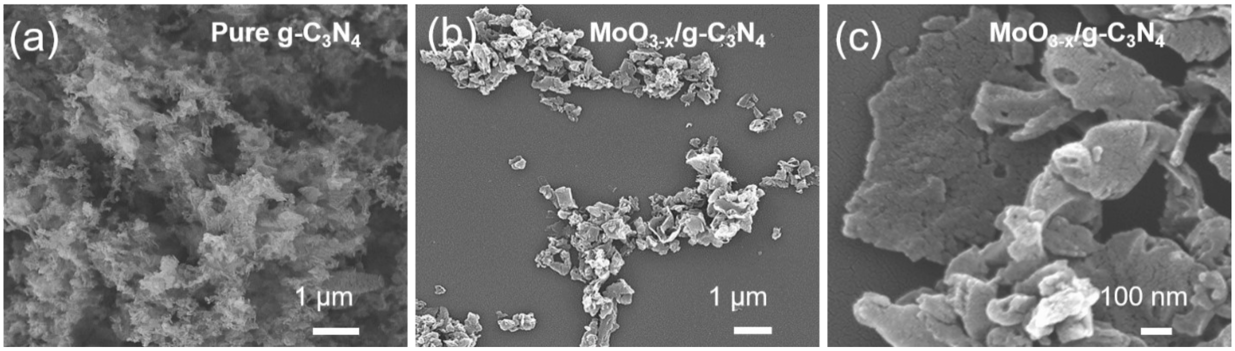

Figure S3 SEM images of pure *g*-C_3N_4 and MoO_{3-x}/*g*-C_3N_4.

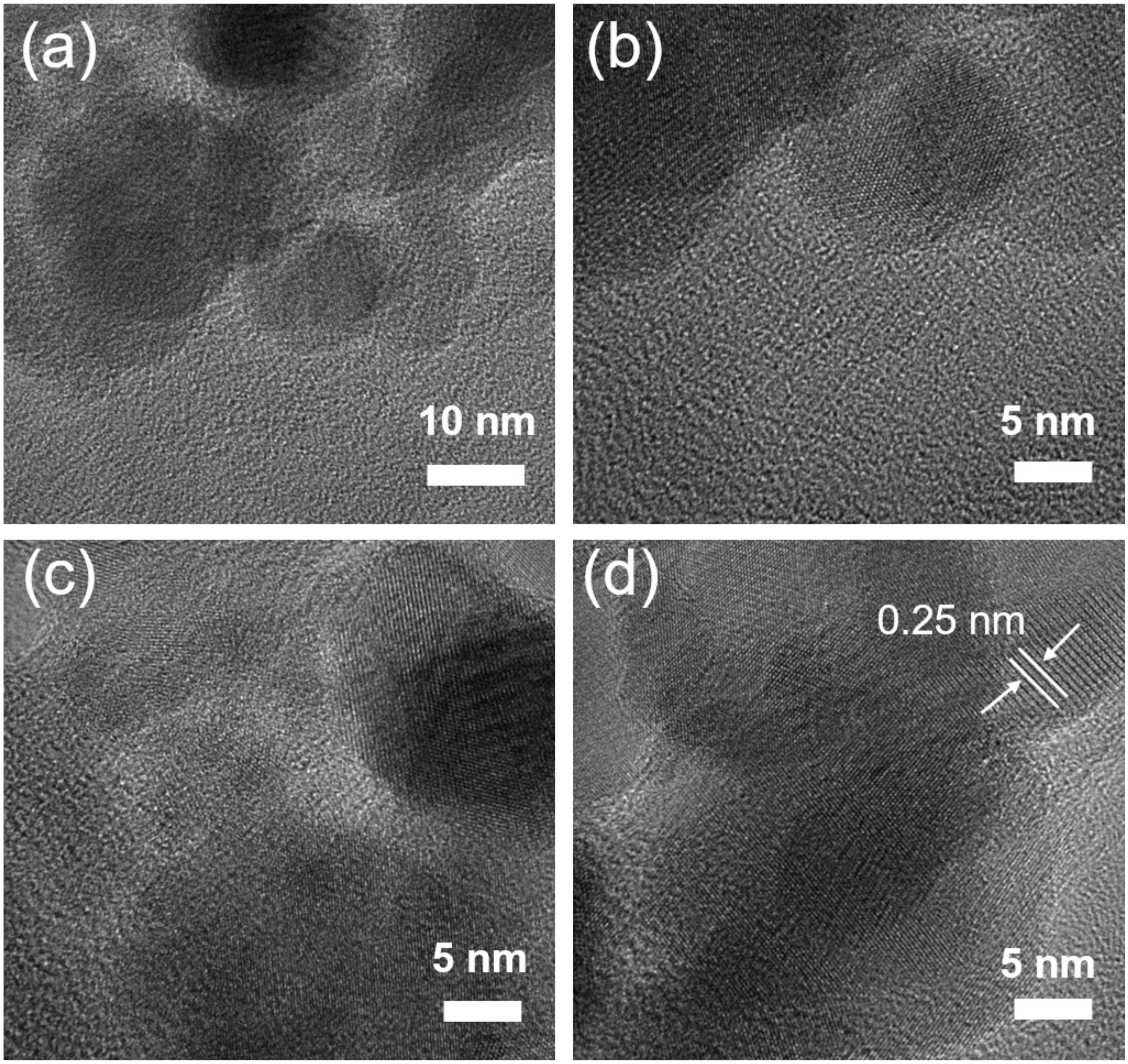

Figure S4 HR-TEM images of MoO_{3-x}/*g*-C_3N_4.

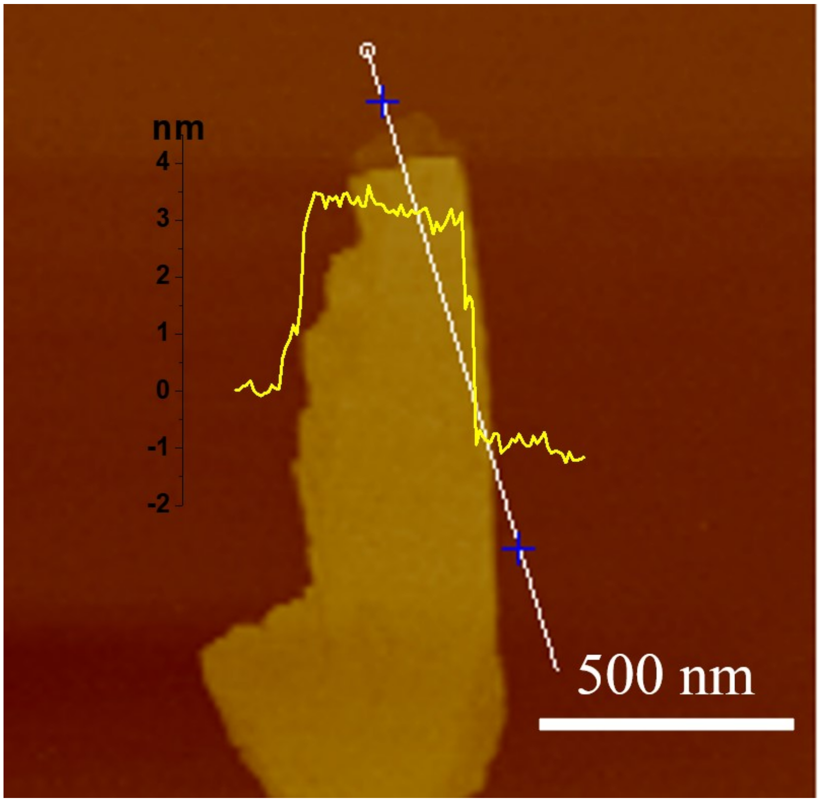

Figure S5 AFM image of pure g-C_3N_4 nanosheets.

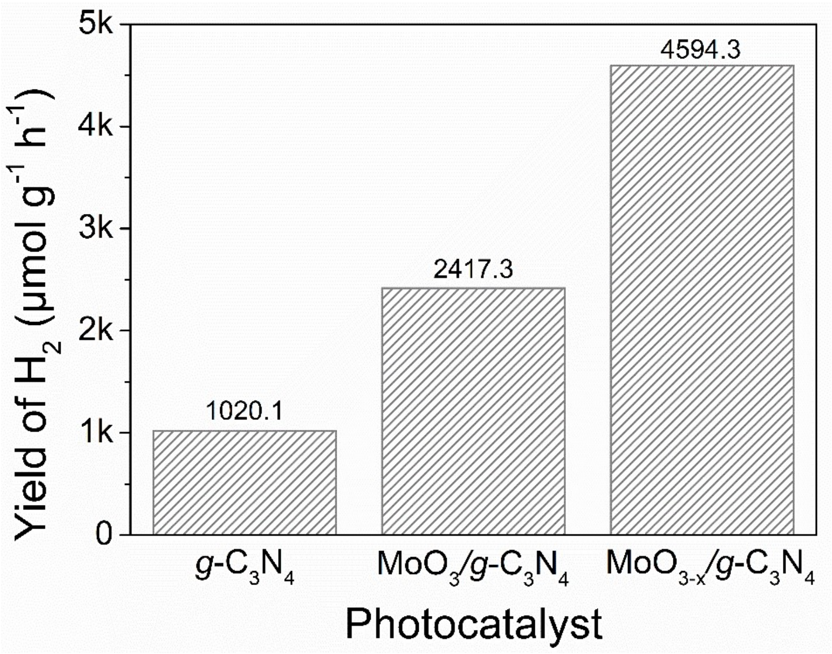

Figure S6 Photocatalytic hydrogen production rates of g-C_3N_4, 5% MoO_3/g-C_3N_4 and 5% MoO_{3-x}/g-C_3N_4.

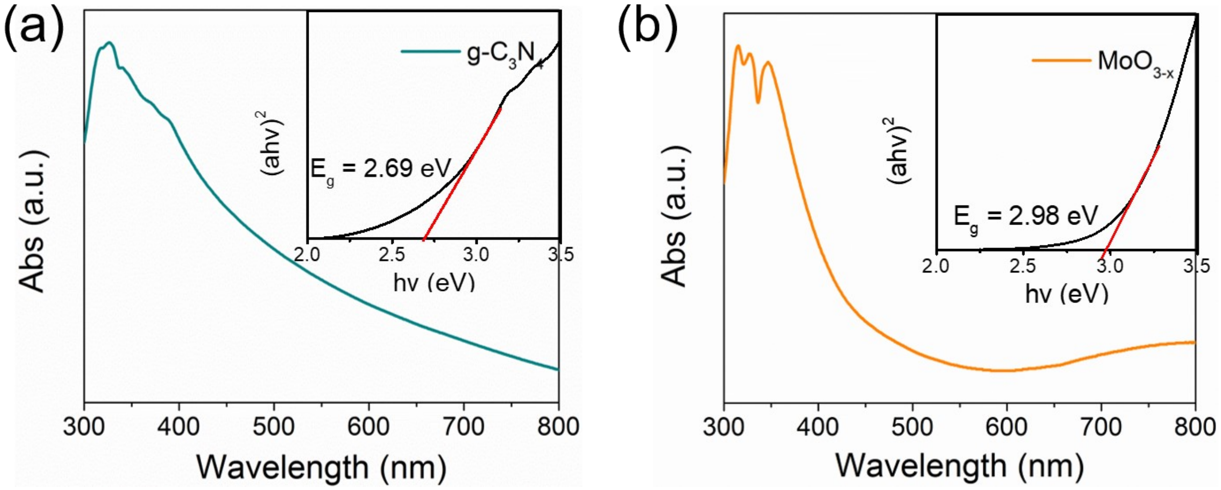

Figure S7 UV−vis diffuse reflectance spectra of *g*-C_3N_4 (a) and MoO_{3-x} (b), the illustrations are their corresponding Tauc's plot.

Table S1. The N/C ratios of g-C_3N_4 and MoO_{3-x}/g-C_3N_4 calculated by XPS.

g-C_3N_4	Atomic %	N/C ratio	MoO_{3-x}/g-C_3N_4	Atomic %	N/C ratio
N1s	54.06		N1s	45.40	
O1s	4.92	1.32	O1s	4.47	1.02
C1s	41.02		C1s	44.08	
			Mo 3s	3.44	
			Na 1s	1.42	
			Cl 2p	1.18	

Table S2. The ratios of different O species of MoO_3 calculated by XPS.

Species	Peak BE	FWHM eV	Area (P) CPS. eV	Ratio %
Lattice oxygen	530.5	1.34	230460.80	72.02
Defect oxygen	532.2	1.67	69881.18	21.84
Hydroxyl oxygen	533.0	1.37	19636.34	6.14

Table S3. The ratios of different O species of MoO_{3-x}/*g*-C_3N_4 calculated by XPS.

Species	Peak BE	FWHM eV	Area (P) CPS.eV	Ratio %
Lattice oxygen	531.1	1.55	17656.19	31.67
Defect oxygen	532.2	1.49	27133.34	48.67
Adsorbed water	535.4	3.16	10963.82	19.66

Table S4. Photocatalytic hydrogen production performance of similar photocatalysts reported in recent references.

Photocatalysts	HER performance /μmol h^{-1} g^{-1}	Co-catalyst	Sacrificial agent	Reference
**MoS_2/*g*-C_3N_4**	3570.0	2 wt% Pt	TEOA	Ref. 1
**WO_3/*g*-C_3N_4**	982.0	2 wt% Pt	lactic acid	Ref. 2
**Cu $(OH)_2$/*g*-C_3N_4**	48.7	--	CH_3OH	Ref. 3
**MoO_{3-x}/*g*-C_3N_4**	4694.3	2 wt% Pt	TEOA	This work
**MoO_{3-x}/*g*-C_3N_4**	821.0	2 wt% Pt	lactic acid	This work

Refences

1. X. Liu, B. F. Wang, M. Liu, S. L. Liu, W. Chen, L. Gao and X. Y. Li, *Appl. Surf. Sci.*, 2021, **554**, 149617.

2. J. Fu, Q. Xu, J. Low, C. Jiang and J. Yu, *Appl. Catal. B*, 2019, **243**, 556-565.

3. X. S. Zhou, Z. H. Luo, P. F. Tao, B. Jin, Z. J. Wu and Y. S. Huang, *Mater. Chem. Phys.*, 2014, **143**, 1462-1468.
